# Severe Hyponatremia Secondary to Ciprofloxacin-Induced Syndrome of Inappropriate Antidiuretic Hormone Secretion (SIADH): A Case Report

**DOI:** 10.7759/cureus.102718

**Published:** 2026-01-31

**Authors:** Ricardo Velho, Manuel Maia, Bernardo Belchior, Leandro Valente, João Coelho, José Brito, Ricardo Geraldes, João Miguel Peixoto, Diogo Leal, Ana Sofia Teixeira

**Affiliations:** 1 Internal Medicine, Unidade Local de Saúde de Coimbra, Coimbra, PRT

**Keywords:** adverse drug reaction, ciprofloxacin, fluoroquinolones, hyponatremia, syndrome of inappropriate antidiuretic hormone secretion (siadh)

## Abstract

Hyponatremia is a common electrolyte disturbance characterized by reduced serum sodium levels, which, when severe, can lead to life-threatening complications. Severe hyponatremia requires prompt treatment and thorough investigation in order to identify the underlying cause and correct the precipitating factor. The syndrome of inappropriate antidiuretic hormone secretion (SIADH) is a cause of euvolemic hyponatremia, involving excessive release of antidiuretic hormone, which promotes renal water reabsorption. Ciprofloxacin, a fluoroquinolone antibiotic, is a rare cause of SIADH through the activation of central nervous system receptors involved in the antidiuretic hormone secretion. The authors report a case of an 84-year-old male with advanced dementia who presented to the emergency department with severe hyponatremia (serum sodium of 103 mmol/L). Diagnostic criteria for SIADH were fulfilled, and after exclusion of other causes of hyponatremia, recent ciprofloxacin exposure was recognized as the cause. Ciprofloxacin was discontinued, and treatment with hypertonic saline and fluid restriction resulted in gradual normalization of serum sodium levels without recurrence. A Naranjo score of 7 supported a probable adverse drug reaction to ciprofloxacin. This case highlights the importance of a systematic medication review in patients with severe hyponatremia and emphasizes that ciprofloxacin, despite being widely prescribed, can rarely induce life-threatening SIADH, warranting increased clinical awareness and prompt drug discontinuation.

## Introduction

Hyponatremia is an electrolyte abnormality defined by low serum sodium levels. Severe hyponatremia may result in serious neurological complications, including seizures and coma, and therefore constitutes a medical emergency. However, even profound hyponatremia may present with minimal or no clinical symptoms [1]. Hyponatremia can be caused by hypovolemic conditions (gastrointestinal losses, diuretics, and adrenal insufficiency), euvolemic conditions (syndrome of inappropriate antidiuretic hormone secretion (SIADH), hypothyroidism, and psychogenic polydipsia), or hypervolemic conditions (heart failure, liver cirrhosis, and nephrotic syndrome). Moreover, pseudohyponatremia can occur in the context of hyperlipidemia, hyperproteinemia, and severe hyperglycemia [2].

The SIADH is a disorder characterized by non-physiological release of antidiuretic hormone. Produced by the hypothalamus, one of the roles of this hormone is the regulation of water reabsorption in the kidneys, inducing the synthesis of water transport proteins (aquaporins) and increasing water reabsorption in the renal collecting duct. This leads to impaired renal water excretion, euvolemic hyponatremia, and inappropriately concentrated urine [3].

Drug-induced SIADH represents an important and potentially reversible cause of hyponatremia, particularly in hospitalized and elderly patients. Commonly implicated medications include antidepressants, antipsychotics, anticonvulsants, and certain chemotherapeutic and antimicrobial agents. Ciprofloxacin, a fluoroquinolone antibiotic, is an exceedingly rare cause of SIADH and, consequently, hyponatremia, with about 10 cases reported worldwide [4]. Despite its rarity, fluoroquinolone-induced SIADH is clinically relevant due to the widespread use of these antibiotics and the potential for severe, life-threatening hyponatremia, particularly in vulnerable populations. In vitro studies suggest that the antibiotic's lipophilic properties enable it to cross the blood-brain barrier, inhibiting the gamma-aminobutyric acid (GABA) receptors and stimulating the N-methyl-D-aspartate (NMDA) receptors, which are implicated in the synthesis and secretion of antidiuretic hormone [5]. Compared with previously published cases, this report describes an exceptionally severe presentation in a very elderly patient, in whom advanced dementia may have obscured the typical manifestations of profound hyponatremia.

## Case presentation

Our report describes an 84-year-old institutionalized, fully dependent for activities of daily living due to advanced dementia, who was brought to the emergency department due to acute dyspnea and hypoxemia (SpO_2_ 75% on room air). On admission, the patient was prostrated, tachypneic, with harsh breath sounds and scattered rhonchi on lung auscultation. His blood pressure and heart rate were normal; no fever was detected. He had no signs of hypervolemia, with no peripheral edema or jugular venous distension. His medical history was significant for advanced dementia with behavioral disturbances requiring chronic antipsychotic therapy and a recent prolonged hospitalization for aspiration pneumonia. There was no history of heart failure, chronic kidney disease, liver disease, or malignancy. There was also no relevant family history of endocrine or renal disease, and no history of alcohol misuse or excessive water intake. Chronic medications included antihypertensive and lipid-lowering agents, as well as multiple psychotropic drugs for behavioral symptoms of dementia. Relevant medications with a known association with hyponatremia included mirtazapine, olanzapine, and risperidone, all of which had been used at stable doses for several months without prior electrolyte disturbances, including during a recent hospitalization. Eight days prior to admission, ciprofloxacin was initiated at a dose of 500 mg twice daily, administered orally, representing the only recent medication change. Initial laboratory results revealed severe hyponatremia (103 mmol/L) and hyposmolality, with high urinary sodium concentration and inappropriately concentrated urine, as depicted in Table 1.

**Table 1:** Initial laboratory results on admission, including urinary spot sodium and osmolality levels. L: liter; kg: kilogram; mmol: millimoles; mOsm: milliosmoles

Parameter	Results on admission	Normal range
Serum Sodium	103 mmol/L	135-145 mmol/L
Serum Osmolality	221 mOsm/kg	260-302 mOsm/kg
Urinary Sodium	96 mmol/L	20-90 mmol/L
Urinary Osmolality	236 mOsm/kg	300-900 mOsm/kg

**Figure 1:**
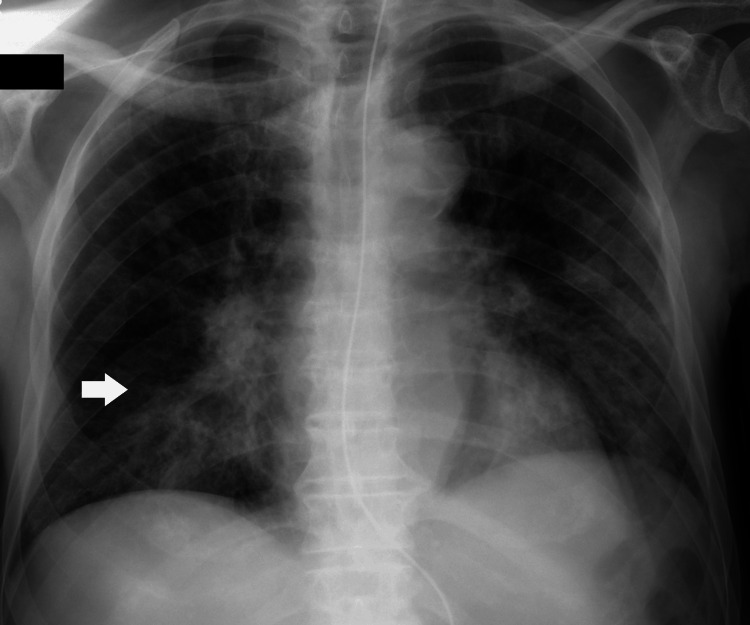
Chest radiograph showing a right lower lung zone consolidation, consistent with pneumonia (arrow).

**Figure 2:**
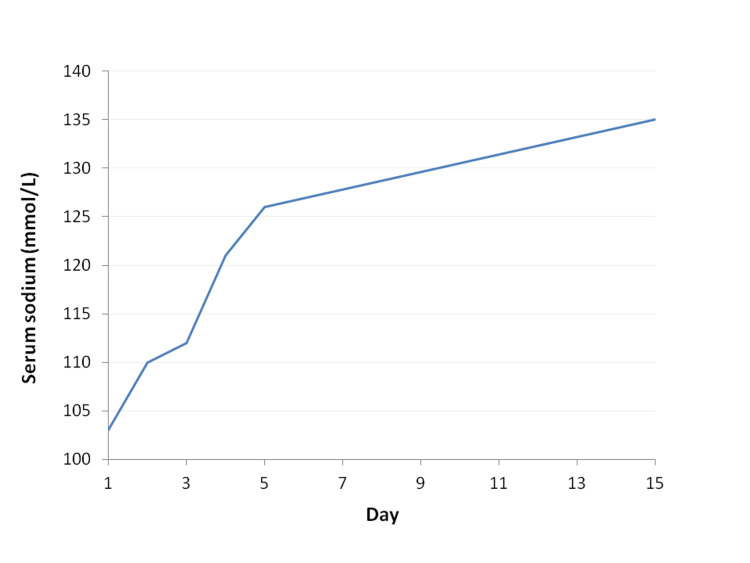
Trendline showing the evolution of serum sodium levels during hospitalization (day 1 = admission). L: liter; mmol: millimoles Ciprofloxacin was discontinued and treatment with hypertonic saline and fluid restriction was initiated on admission.

The remaining laboratory and imaging tests on admission confirmed the diagnosis of pneumonia (Figure 1), which was causing respiratory failure in a patient with a recent hospitalization due to aspiration pneumonia. During that previous hospitalization, serum sodium levels remained within the normal range. Following discharge, the patient was treated with ciprofloxacin for worsening respiratory symptoms. No other medications had been initiated or dose-adjusted, and all chronic therapies had remained stable. Further laboratory studies showed normal glucose levels and normal renal function. Thyroid and adrenal function were evaluated during admission and were within normal limits, with normal thyroid-stimulating hormone and free thyroxine levels, as well as normal morning cortisol levels. The diagnosis of SIADH was supported by the presence of severe hypotonic hyponatremia in a clinically euvolemic patient, with inappropriately concentrated urine and elevated urinary sodium levels. Renal function was normal, as were thyroid and adrenal function tests, and there was no history of recent diuretic use. Other causes of hyponatremia were systematically excluded. Upon admission, ciprofloxacin was discontinued, and treatment for hyponatremia was initiated, including water restriction and hypertonic saline administration. Serum sodium levels gradually normalized, and no further episodes of hyponatremia occurred during hospitalization, as depicted in Figure 2.

Regarding the pneumonia that was diagnosed on admission, multidrug-resistant *Klebsiella pneumoniae *was identified in sputum cultures, and the patient was treated with intravenous meropenem, with progressive recovery to baseline. He was discharged home after 15 days of hospitalization. Blood sodium levels were evaluated one month after discharge, and the patient had normal sodium levels. A summary of key clinical events is presented in Table 2. 

**Table 2:** Timeline of key clinical events. L: liter; mmol: millimoles

Timepoint	Key events
Baseline	Institutionalized elderly male with advanced dementia; chronic stable medication; normal serum sodium during recent hospitalization
Day 8	Initiation of oral ciprofloxacin for respiratory symptoms
Day 0	Emergency department admission for dyspnea and hypoxemia; severe hyponatremia (serum sodium 103 mmol/L)
Hospital course	Discontinuation of ciprofloxacin; fluid restriction and hypertonic saline; treatment of pneumonia
Outcome	Gradual normalization of serum sodium; no recurrence; discharged after 15 days
Follow-up	Normal serum sodium one month after discharge

## Discussion

This case describes a rare but potentially life-threatening adverse effect of ciprofloxacin: SIADH resulting in severe hyponatremia. Although fluoroquinolones are generally considered safe and well-tolerated, clinicians should be aware that, in rare cases, they may precipitate clinically significant electrolyte disturbances.

The proposed mechanism underlying fluoroquinolone-induced SIADH involves central nervous system effects. Due to their lipophilic properties, fluoroquinolones are able to cross the blood-brain barrier and interfere with neurotransmitter pathways, including inhibition of GABA receptors and stimulation of NMDA receptors. This imbalance may lead to hypothalamic hyperexcitability and inappropriate secretion of antidiuretic hormone, resulting in water retention and dilutional hyponatremia [4-7].

Ciprofloxacin-induced SIADH is exceedingly rare, with only a limited number of cases reported worldwide. Similar cases have also been described with other fluoroquinolones, including levofloxacin and moxifloxacin [8-10]. Reported cases predominantly involve elderly patients and typically describe the development of hyponatremia shortly after drug initiation, with resolution following drug withdrawal. The present case is distinguished by the extreme severity of hyponatremia and the presence of advanced dementia, which may have masked classical neurological manifestations and delayed recognition.

Pulmonary infections, including pneumonia, are a recognized cause of SIADH and represent a potential confounder in this case. However, during a recent prolonged hospitalization for aspiration pneumonia, this patient maintained normal serum sodium levels. Hyponatremia developed only after the initiation of ciprofloxacin and resolved completely following its discontinuation, despite persistence of pulmonary infection and the need for alternative antibiotic therapy. This temporal relationship strongly supports ciprofloxacin as the primary trigger of SIADH in this patient.

Given these considerations, a structured differential diagnosis for hyponatremia was considered in this case. Hypovolemic hyponatremia was deemed unlikely given the absence of gastrointestinal losses, clinical euvolemia, elevated urinary sodium concentration, and lack of response suggestive of volume depletion. Hypervolemic causes, including heart failure, liver cirrhosis, and nephrotic syndrome, were excluded based on clinical examination and the absence of relevant medical history. Endocrine causes such as hypothyroidism and adrenal insufficiency were ruled out by normal thyroid and adrenal function tests. Drug-induced hyponatremia from chronic psychotropic medications was considered; however, these agents had been used at stable doses for several months without prior electrolyte disturbances, including during recent hospitalization. In contrast, ciprofloxacin was the only newly introduced medication, with a clear temporal association and resolution following drug withdrawal.

The diagnosis of SIADH was supported by hypotonic hyponatremia in a clinically euvolemic patient, with inappropriately concentrated urine and elevated urinary sodium levels, as well as normal renal, thyroid, and adrenal function. The Naranjo adverse drug reaction probability scale yielded a total score of 7, corresponding to a probable adverse drug reaction [11]. The score was primarily driven by the clear temporal relationship, objective laboratory confirmation, exclusion of alternative causes, and improvement following drug withdrawal, while rechallenge and dose-response assessment were not applicable in this case (Table 3).

**Table 3:** Naranjo adverse drug reaction probability scale applied to the present case.

Naranjo item	Question	Score	Evidence from this case
1	Are there previous conclusive reports on this reaction?	+ 1	Ciprofloxacin-induced SIADH has been previously reported in the literature
2	Did the adverse event appear after the suspected drug was administered?	+ 2	Severe hyponatremia developed after initiation of ciprofloxacin
3	Did the adverse reaction improve when the drug was discontinued?	+ 1	Serum sodium normalized after ciprofloxacin withdrawal
4	Did the adverse reaction reappear when the drug was readministered?	0	Rechallenge was not performed
5	Are there alternative causes that could have caused the reaction?	+ 2	Other causes were systematically excluded
6	Did the reaction reappear when a placebo was given?	0	Not applicable
7	Was the drug detected in blood or other fluids in toxic concentrations?	0	Drug levels were not measured
8	Was the reaction more severe when the dose was increased or less severe when the dose was decreased?	0	Dose changes were not performed
9	Did the patient have a similar reaction to the same or similar drugs in the past?	0	No previous similar reactions reported
10	Was the adverse event confirmed by any objective evidence?	+ 1	Objective laboratory findings confirmed SIADH

From a clinical and pharmacovigilance perspective, this case highlights the importance of careful medication review in patients presenting with severe hyponatremia, particularly among elderly and cognitively impaired individuals. Even widely prescribed antibiotics such as ciprofloxacin may rarely precipitate serious adverse effects, and early recognition with prompt discontinuation can be life-saving.

## Conclusions

Ciprofloxacin-associated SIADH is a rare but potentially life-threatening condition that clinicians should be aware of, particularly when evaluating severe hyponatremia in elderly patients. While definitive causality cannot be established from a single case, the present report adds to existing evidence by illustrating a likely ciprofloxacin-associated SIADH with severe hyponatremia, reinforcing the need for clinical awareness and pharmacovigilance. The temporal association, exclusion of alternative causes, and resolution after drug discontinuation support a probable adverse drug reaction in this patient.

This case highlights several important clinical learning points, including the need for careful medication review in patients presenting with hyponatremia, increased awareness of fluoroquinolone-associated electrolyte disturbances, and the importance of monitoring serum sodium levels in high-risk populations, such as elderly and cognitively impaired individuals. Early recognition and prompt withdrawal of the suspected offending agent may prevent serious complications.
